# Fragile Sites of ‘Valencia’ Sweet Orange (*Citrus sinensis*) Chromosomes Are Related with Active 45s rDNA

**DOI:** 10.1371/journal.pone.0151512

**Published:** 2016-03-15

**Authors:** Hong Lan, Chun-Li Chen, Yin Miao, Chang-Xiu Yu, Wen-Wu Guo, Qiang Xu, Xiu-Xin Deng

**Affiliations:** 1 Key Laboratory of Horticultural Plant Biology (Ministry of Education), Huazhong Agricultural University, Wuhan, 430070, China; 2 College of Life Science and Technology, Huazhong Agricultural University, Wuhan, 430070, China; Huazhong university of Science and Technology, CHINA

## Abstract

*Citrus sinensis* chromosomes present a morphological differentiation of bands after staining by the fluorochromes CMA and DAPI, but there is still little information on its chromosomal characteristics. In this study, the chromosomes in ‘Valencia’ *C*. *sinensis* were analyzed by fluorescence *in situ* hybridization (FISH) using telomere DNA and the 45S rDNA gene as probes combining CMA/DAPI staining, which showed that there were two fragile sites in sweet orange chromosomes co-localizing at distended 45S rDNA regions, one proximally locating on B-type chromosome and the other subterminally locating on D-type chromosome. While the chromosomal CMA banding and 45S rDNA FISH mapping in the doubled haploid line of ‘Valencia’ *C*. *sinensis* indicated six 45S rDNA regions, four were identified as fragile sites as doubled comparing its parental line, which confirmed the cytological heterozygosity and chromosomal heteromorphisms in sweet orange. Furthermore, Ag-NOR identified two distended 45S rDNA regions to be active nucleolar organizing regions (NORs) in diploid ‘Valencia’ *C*. *sinensis*. The occurrence of quadrivalent in meiosis of pollen mother cells (PMCs) in ‘Valencia’ sweet orange further confirmed it was a chromosomal reciprocal translocation line. We speculated this chromosome translocation was probably related to fragile sites. Our data provide insights into the chromosomal characteristics of the fragile sites in ‘Valencia’ sweet orange and are expected to facilitate the further investigation of the possible functions of fragile sites.

## Introduction

Sweet orange (*Citrus sinensis* [L.] Osbeck), as the most important *Citrus* species with great economic and health value, is grown in 114 countries and accounts for nearly 70% of the world’s *Citrus* production [1]. *C*.*sinensis* is believed to arise from the crossing between ancestral pommelo and mandarin with polymorphic alleles in the genome [2]. The breeding and related researches of *C*. *sinensis* are rather challenging because of its high heterozygosity [3]. Doubled haploid lines of ‘Valencia’ sweet orange (*C*. *sinensis* [L.] Osbeck) has been generated via anther culture [4], which carrying a gametophytic chromosome constitution is extremely valuable for genetic and genomic studies. Then further clarifying the chromosome characteristics of *C*. *sinensis* combining with its double haploid line is essential for understanding the genome structure of sweet orange from chromosomal view. Although the *Citrus* chromosome number (2n = 2x = 18) is relatively small, it is challenging to obtain a fine karyotype with traditional cytology methods, because *Citrus* metaphase chromosomes are very small and morphologically similar. The employment of chromomycin A3 (CMA) and 4′, 6-diamidino-2-phenylindole (DAPI) present a remarkable differentiation of bands and has allowed the identification of several chromosomal types. According to the distribution and number of the CMA bands, Carvalho et al. [5] defined eight types as A, B, C, D, E, F, FL and G, modified on the report by Guerra [6]. The D-type and F-type are common in the majority of *Citrus* chromosomes, whereas the A, B, C, E, and G types are relatively rare and therefore more meaningful for karyotyping [7, 8]. *In situ* hybridization techniques, such as fluorescence *in situ* hybridization (FISH) and genomic *in situ* hybridization (GISH), the most reliable methods for cytogenetic studies, are very powerful for identifying chromosome characterization and genome organization [9]. Invariable and heterozygotic karyotype among sweet orange cultivars were revealed by CMA/DAPI staining and *in situ* hybridization and the putative hybrid origins of these species from mandarin (*C*. *reticulata*) and pummelo (*C*. *grandis*) were inferred [10]. CMA banding and BACs-FISH can generate unique signal patterns to map the chromosomes of *Poncirus trifoliata* and to provide comparative cytogenetic maps of *Citrus* and trifoliate orange for karyotyping and phylogenetic analysis [11–14].

The ribosomal RNA genesloci are essential housekeeping genes, and abundance of rDNA maintains genome integrity [15, 16]. 45S rDNA loci comprising 18S-5.8S-26S generally refer to the nucleolar organizer regions (NORs) and become effective cytological markers for karyotype analysis, especially in species with many small and similar chromosomes because it is highly conserved and located in one or more clusters of chromosomes [17]. The plants have more copies of rDNA than necessary, which vary in the number and location between intra- and interspecific variation, and some of them don’t have transcriptional activity. 45 rDNA in many accessions of *Citrus* has been investigated by Marques et al [18], which showed that the number of 45S rDNA varies from two to five. Furthermore, transcriptionally active decondensed 45S rDNA sites in *Citrus* were found to be DNA hypomethylated, while the silenced condensed sites were strongly 5-mCyt methylated [18]. Originally from human chromosome research fragile sites were defined as the regions that are especially prone to forming non-staining gaps, constrictions or breaks in one or both of the chromatids on metaphase chromosomes [19]. In human chromosomes, 45S rDNA sites of X chromosomes occasionally appeared an axial distribution and lateral expansions under confocal laser scanning microscopy, and presented a lightly stained chromatin structure [20]. This type of 45S rDNA units were considered to correspond to the most fragile sites containing hotspots of DNA double-strand breaks [21]. Under DNA replication stresses, such as treatment with low level of DNA polymerase alpha, these sites are prone to break and the mitotic recombination in *Saccharomyces cerevisiae* is increased [22]. Such breakages are frequently involved in chromosomal rearrangements in cancer cells and play an important role in human oncogenesis [23, 24]. Chromatin epigenetic modifications such as DNA methylation, histone methylation and acetylation are associated with the changement of chromatin structure and related to the expression of fragile sites [25, 26]. It has been reported that chromosome gaps, constrictions and breaks in *Lolium* were observed at a high frequency on mitotic chromosomes by atomic force microscope (AFM), and these fragile sites were exclusively associated with the 45S rDNA which resulted in chromosome fragmentation in diploid and tetraploid [27–30]. When aphidicolin or actinomycin D treatment in several plant species, 45S rDNA fragile phenotypes appeared, and epigenetic alterations was observed such as DNA methylation and level of histone H3 decreasing, and histone acetylation and level of H3K4me2 increasing with the 45S rDNA fragile regions accompanied [30].

So far, chromosome fragile sites in woody plants have not been reported, although lightly–staining chromatin depletion decondensation occurred at 45S rDNA regions are common in *Citrus* and related genera [8, 31–35]. In the present paper, we employed the doubled haploid callus line of *C*. *sinensis* [L.] Osbeck as the control to investigate the chromosomal characteristics of ‘Valencia’ sweet orange. By combining CMA/DAPI banding with repetitive DNA FISH, the cytological heterozygosity, and the number and loci of the fragile sites in ‘Valencia’ sweet orange were revealed. Furthermore, Ag-NOR showed that the distended 45S rDNA regions as fragile sites had transcriptional activity. The number and characteristics of fragile sites in *Citrus* were proposed and their possible functions were discussed.

## Materials and Methods

### Plant materials and chromosome preparation

Fruits of ‘Valencia’ sweet orange cv. Rohde Red (*C*. *sinensis* [L.] Osbeck) were obtained from the National Center of Citrus Breeding, Huazhong Agricultural University, Wuhan, China. Seeds were peeled off and cultivated in pots in the incubator at 28°C. Root tips from the germinated seeds (about 3–5 mm long) were successively collected. The doubled haploid callus line from anther culture of ‘Valencia’ orange was suspension-cultured to be vigorous. To harvest metaphase cells, young tissues were obtained and pretreated in a saturated aqueous solution of P-dichlorobenzene for 2 h at 20°C and fixed in 3:1 ethanol/glacial acetic acid (v/v) for 24 h, and then were stored at -20°C in 70% ethanol solution until use. Rinsed tissues were macerated in an enzyme mixture containing 0.25% pectinase (Sigma), 0.25% pectolyase Y-23 (Yakult) and 0.5% cellulose RS (Onozuka) for 80–90 min at 37°C. Then, the meristem was mashed using a fine needle with a drop of 60% acetic acid, and the slide was flame-dried with a drop of fixation solution.

### FISH and CMA/DAPI staining

Plasmid 45S rDNA kindly provided by Kang et al. [36] was isolated by alkaline lyses. The plasmid DNA was labeled with biotin 16-dUTP (Roche) using the Nick Translation kit (Roche). (5’-CCCTAAA-3’)_5_, which was used as a telomeric marker probe, was synthesized with digoxigenin at 5’ end by Shanghai invitrogen trading Co, Ltd. (China). FISH was performed following the procedures of Cui et al. [37]. The immune-detection of biotinylated and digoxigenated DNA probes was carried out by using Cy3-labeled streptavidin (Sigma) and anti-digoxigenin conjugate-FITC (Roche), respectively. Slides were counterstained with a mixing solution in a proportion of 1:1 (v/v)(4 μg/ml 4’-6-diamidino-2-phenylindole (Roche):5 mg/ml chromomycin A3(Sigma)), and were mounted in antifade solution of Vectashield (Vector Laboratories, Peterborough, UK) for 2 h at room temperature. Slides were stored over-night at 4°C to stabilize the fluorochromes, and then were examined under a Zeiss Scope A1 fluorescence microscope (Zeiss, Germany) equipped with a Hamamatsu digtal camera. Images were processed by Adobe Photoshop 8.0 software (Adobe Systems, San Jose, CA, USA) to combine or to adjust contrast and brightness. Separate monochrome images were captured for chromosomes (DAPI) or 45S rDNA (Cy3), and then converted into blue and green images, respectively. Kymograms were recorded by using the “linescan” command in the software MetaMorph 4.6.3 with the DAPI and 45S rDNA fluorescence signal intensity as key parameters.

### Silver staining of nucleolar organizing regions (NORs)

50 μl 50% (W/V) silver nitrate was added to each slide with the spreading of chromosomes and nuclei. The slides were incubated in a moist chamber at 70°C for 1 h and washed with distilled water, and then were air-dried. The slides were covered with a mixing staining solution in a proportion of 1:1 (v/v)(0.4g/ml AgNO_3_ in 50% NH_4_OH: 0.5% formic acid)and observed under a bright field microscope (Olympus BX61) until the NORs turned black. After staining, the slides were washed with running deionized water and air-dried again. Statistical analysis on the number of nucleoli and NORs were conducted in more than 100 interphase cells and 10 metaphase cells.

### Meiotic analysis of Valencia sweet orange

Appropriate young flower buds of ‘Valencia’ sweet orange at various developmental stages were collected in the morning (9:00–10:00 AM), and immediately fixed in 5:3:2 ethanol: glacial acetic acid: chloroform for 24h at room temperature. The anther were taken out and rinsed, then macerated in an enzyme mixture containing 0.25% pectinase (Sigma), 0.25% pectolyase Y-23 (Yakult) and 0.5% cellulose RS (Onozuka) for 60–70 min at 37°C. Microsporocytes were squeezed out of anthers and stained in 1% aceto-carmine on a clean microscope slide. The preparations were observed using a microscope (BX61: Olympus), and photos were taken with an attached video camera (DP70: Olympus). At least 30 PMCs late diakinesis used to analyze the characteristics of meiotic chromosome paring were observed, and the numbers of chromosome configurations per PMC were recorded.

### Cytological measurement and analysis

Metaphase of both diploid and dihaploid were analyzed for the position and distribution of CMA-DAPI bands, 45S rDNA, telomeric sites and for the integrity of chromosome morphology. We measured the relative length and arm ratio of the chromosomes. The centromeres were indicated by the positions of the primary constrictions. Measurements were performed on digital images using Image-Pro Plus 6.0 software. The banding patterns were determined on the basis of the numbers and positions of CMA positive bands. The ideogram was prepared using Microsoft Office Excel 2003.

## Results

### Chromosome karyotyping of parental diploid *C*.*sinensis* cv ‘Valencia’ and its doubled haploid line (Dihaploid)

**Fig 1:**
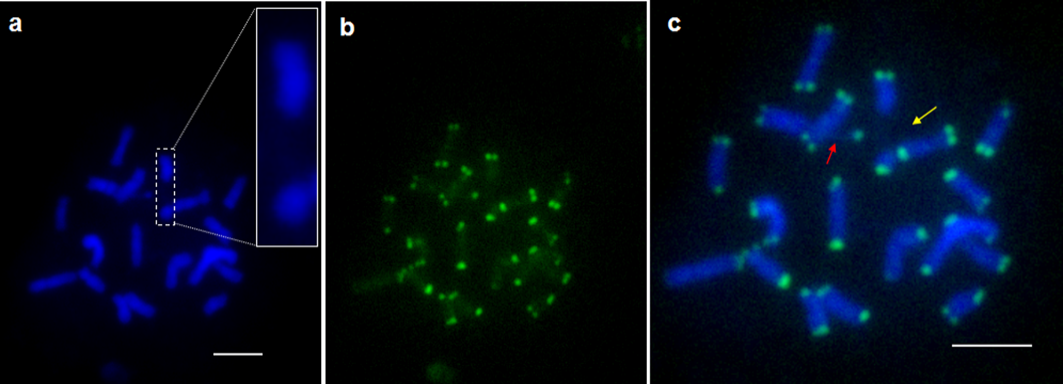
The telomeric sites of metaphase chromosomes in diploid *C*. *sinensis*. a, DAPI-stained chromosomes; b, FISH signals; c, merged images of the DAPI-stained chromosomes and the FISH signals. Yellow arrow indicates the break from the middle of the chromosome and red arrow indicates the break at the subterminal of the chromosome. Break chromosome was enlarged in more detail. Scale bars = 5μm.

Hundreds of metaphase cells were observed in parental diploid *C*. *sinensis* by cytological examination using a routine chromosome preparation procedure. Interestingly, we found that the number of chromosomes plus chromosome fragments was always 19, which was larger than the expected 18 in most mitotic cells, and it is rather different to distinguish whether one “chromosome” is a complete chromosome or merely a chromosome fragment before telomere-FISH (Fig 1A). In order to confirm the chromosome numbers, FISH analysis was conducted with telomere-specific repeated sequences (TTTAGGG)_5_ as a probe mapping to metaphase chromosomes. Detection of telomeric DNA hybridization signals showed that 36 telomere signals were observed in each cell (Fig 1B). All these signals were located at the terminals of chromosomes, and no telomeric signals were detected at the chromosome break and in generated fragments (Fig 1C). The chromosome numbers of the diploid was counted as 18 according to telomeric sites. The telomere-FISH results showed that there were two gaps in each cell of diploid: one at the chromosome subterminal (indicated by red arrow) and the other was proximal on the chromosome (indicated by yellow arrow) (Fig 1C), suggesting that some chromosome fragments had been counted as complete chromosomes before. These interesting chromosome-break phenomena were observed uniformly in most metaphase cells. Cytological appearance of the breakage, gap or constriction in diploid appears to be analogous to that of fragile sites observed in human chromosomes and ryegrass [19–21, 26–30], showing that there are two chromosome fragile sites in diploid.

**Fig 2:**
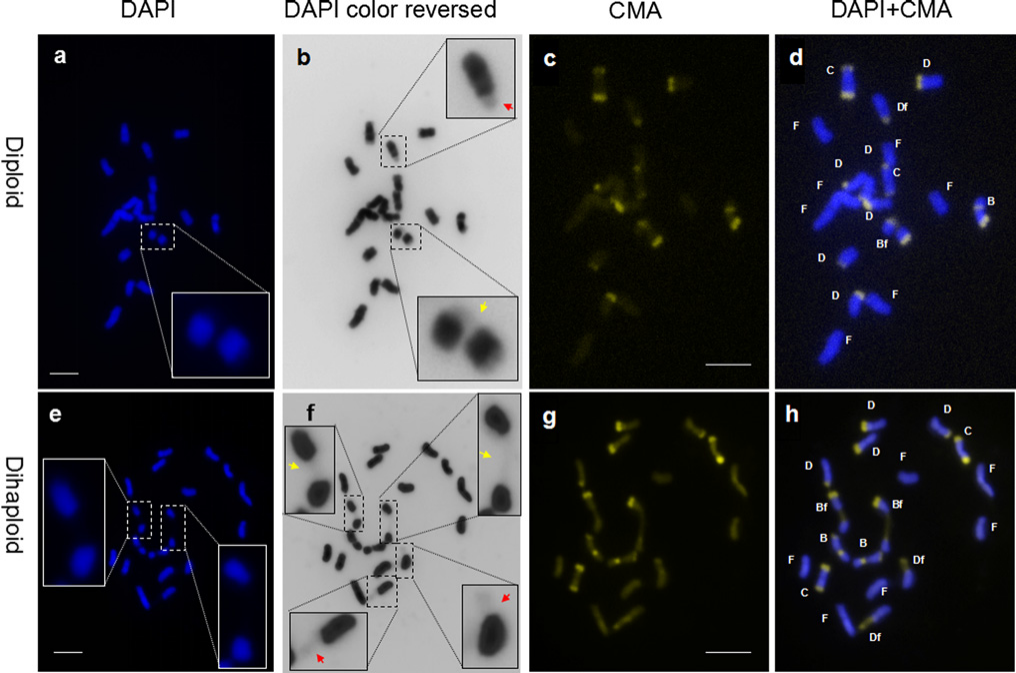
DAPI/CMA doubled fluorescence banding of *C*. *sinensis* (diploid) and its doubled haploid callus line (dihaploid). a and e, DAPI staining chromosomes; b and f, reverse image of image a and e for the visualization of fragile sites in metaphase chromosomes (yellow arrows indicate the proximal fragile sites and the red ones indicate subterminal fragile sites); c and g, CMA staining chromosomes; d and h, merged images of DAPI/CMA staining chromosomes (B, Bf, C, D, Df and F indicate the type of each chromosome).The chromosomes with fragile site are enlarged in more detail. Bar scales = 5μm.

The mitotic chromosomes in *C*. *sinensis* can be cytologically classified into four types, which are respectively named as B (one terminal band and one proximal band), C (two terminal bands), D (one terminal) and F (no band) according to CMA/DAPI banding [6, 33]. In this study, the karyotype formulas based on CMA+ bands were 2B+2C+7D+7F for diploid *C*.*sinensis* cv. Valencia (Fig 2C), and 4B+2C+6D+6F for its dihaploid (Fig 2G). Black-white reversed images of DAPI-stained metaphase chromosomes (Fig 2A and 2E) enhanced the visualization of the proximal fragile sites indicated by yellow arrows and the subterminal ones indicated by red arrows (Fig 2B and 2F). The combined chromosome images of DAPI and CMA staining (Fig 2D and 2H) showed that the proximal fragile sites were located in B-type chromosomes and the subterminal fragile sites were located in D-type chromosomes, and all of them were co-located with CMA+ bands. According to CMA karyotyping and the characteristics of fragile sites, the B-type chromosomes with a proximal fragile sites were renamed as Bf and the type D chromosomes with a subterminal fragile sites were renamed as Df. Hence, the karyotype formulas were modified as B+Bf+2C+6D+Df+7F in diploid *C*. *sinensis* (Fig 2D) and 2B+2Bf+2C+4D+2Df+6F in dihaploid (Fig 2H), respectively.

### Location of 45S rDNA by FISH mapping in parental diploid *C*.*sinensis* cv Valencia and its doubled haploid line (Dihaploid)

**Fig 3:**
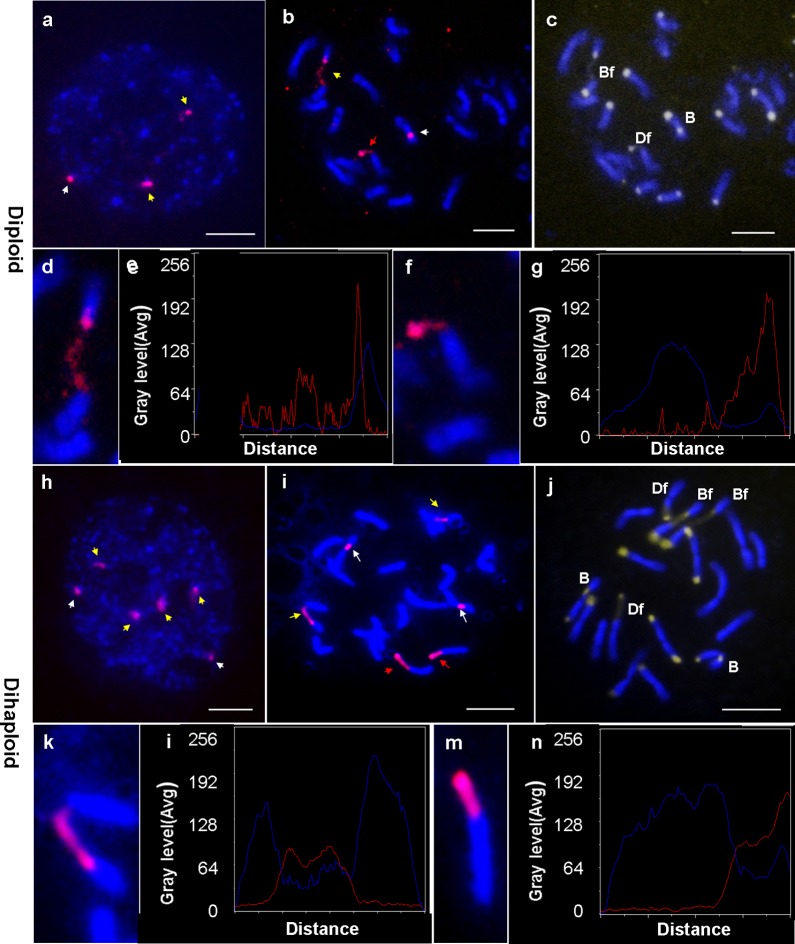
Localization of 45S rDNA by FISH in diploid (a-g) and dihaploid (d-n). a and h, red hybridization signals of 45S rDNA on interphase nucleus stained by DAPI; b and i, red Cy3 as hybridization signals of 45S rDNA on metaphase chromosomes stained by DAPI; c and j, DAPI and CMA staining chromosomes (B, Bf, D and Df indicate the type of each chromosome); d, f, k and m, enlarged images of chromosome with fragile site; e, g, i and n, linescan curve analysis of fluorescence signals for the chromosomes with 45S signals. The horizontal axis is the length of one chromosome, and the vertical axis is the gray level which measures the intensity of the fluorescent dye and signals. The red line represents the hybridization site and signal intensity, and the blue line shows chromosomes stained by DAPI. The yellow arrows indicate proximal decondensed 45S rDNA loci and the red arrows indicate the subterminal decondensed 45S rDNA loci, while white arrows indicate condensed 45S rDNA loci. Bar scales = 5 μm.

In plants as well as in animals, 45S rDNA sites, which are tandem DNA sequences, are important cytological characteristics of chromosomes. Based on the karyotyping and FISH analyses, the number and location of 45S rDNA were indicated on the mitotic interphase nucleus and metaphase chromosomes in diploid *C*. *sinensis* and its dihaploid line. The results showed that three signal sites of 45S rDNA in *C*. *sinensis* and six signal sites in dihaploid line were detected in all examined cells (Fig 3). In *C*. *sinensis*, on the mitotic interphase nucleus, two decondensed sites were at the nucleolus region and one condensed site was at the edge of the nucleus (Fig 3A). On metaphase chromosomes, two of these signals were observed at the proximal CMA+ band of the B-type and Bf-type chromosomes and the third signal was found at the subterminal CMA+ band of the Df chromosome (Fig 3B and 3C). Meanwhile, there were six strong 45S rDNA signals in Dihaploid line (Fig 3H–3J). On the mitotic interphase nucleus, four decondensed sites were at the nucleolus region and two condensed sites were at the edge of the nucleus (Fig 3H). On metaphase chromosomes, two pairs of these sites were observed at the proximal CMA+ band of B-type and Bf-type chromosomes and one pair was found at the terminal CMA+ band of Df chromosomes (Fig 3I and 3J). Linescan curve analysis of fluorescence signals for the chromosomes with 45S rDNA indicated that chromatin depletion or decondensation occurred within the 45S rDNA regions. The chromosomes with fragile site were enlarged and the fluorescence signals were analyzed. In the linescan curve, this difference in signal intensity was reflected by the height of the wave crests, and the red curve and the blue curve were very complementary. In *C*.*sinensis* the occurrence of a sharp rise in the end of the 45S regions in Fig 3D and 3F indicated that a strong fluorescence signal was present only at the end of fragile sites while the line was almost gentle before, suggesting that a few 45S rDNAs remain on this part of the chromosome (Fig 3D–3G). In dihaploid, the relatively flat red linescan curve at high lever in the gap of the chromosome indicated 45S rDNA regions had strong fluorescence and chromatin depletion or decondensation remained within the whole 45S region (Fig 3K–3N). The linescan curve data were in good agreement with the cytological observations provided in Fig 3D, 3F, 3K and 3M. Obviously, all the FISH signals of 45S rDNA on the Bf-type and Df chromosomes were dispersed and chromatin depletion or decondensation occurred in fragile sites.

### Transcriptional activities of 45S rDNA sites by silver staining in ‘Valencia’ sweet orange (*C*.*sinensis*)

**Fig 4:**
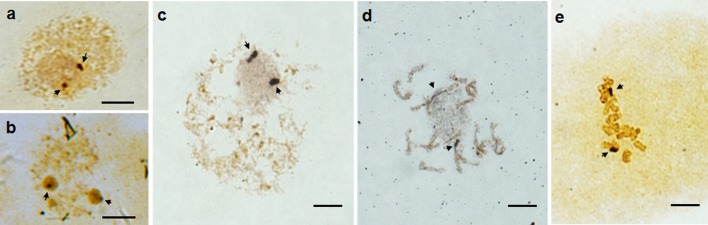
Silver staining of NORs in *C*. *sinensis*. a, interphase nuclei with one nucleolus; b, interphase nuclei with two nucleoli; c, prometaphase cell; d and e, metaphase chromosomes. Arrows indicate silver-stained NORs. Scale bars = 5 μm.

In order to assess the transcriptional activities of these 45S rDNA sites, Ag-NOR staining was conducted. Observations revealed that only two of the three 45S rDNA sites marked by FISH showed Ag-NOR bands in metaphase mitosis of *C*. *sinensis* (Fig 4).The two 45S rDNA sites with gaps showed NOR bands and were active, while the other one was silenced. 97.4% interphase cells (671 in 689) had two condensed Ag-NOR sites at the edge of the nucleolus in each cell (Fig 4A), even in the 2.6% cells (18 in 689) with two nucleoli (Fig 4B). At prometaphase, only one nucleolus was observed to carry two distended Ag-NOR sites near the nucleolar boundary (Fig 4C). At metaphase, there were two distended Ag-NOR sites co-localized with the fragile sites (Fig 4D and 4E).

### Meiosis observation in PMCs of Valencia sweet orange

**Fig 5:**
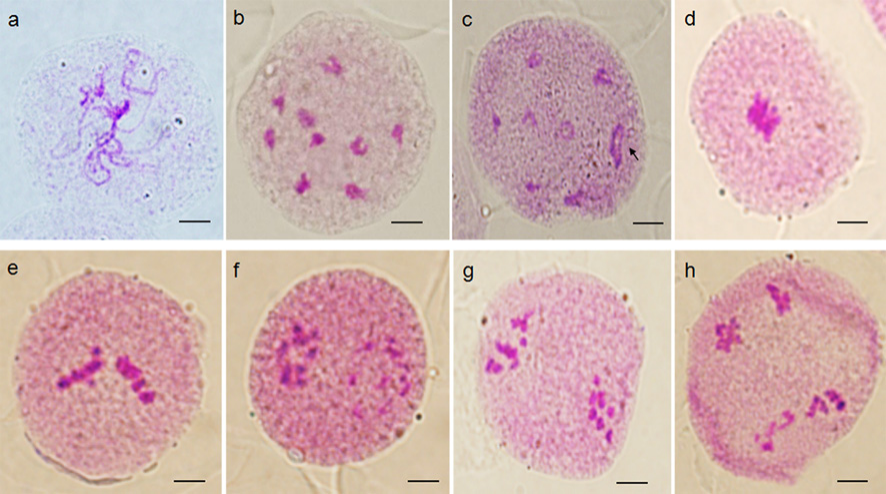
Meiotic analysis of ‘Valencia’ sweet orange. Pachytene(a), diakinesis(b and c), metaphase I (d), anaphase I (e), metaphase II (f), anaphase II (g), and telophase II(h). Chromosome pairing at diakinesis showed 9 bivalents in b, and 7 bivalents + 1 quadrivalent in c. Arrow in c indicates quadrivalent. Bar = 5 μm.

The process of meiosis essentially involves two cycles of division, involving a pollen mother cells (PMCs) dividing and then dividing again to form 4 haploid cells. The representatives PMC meiosis process in ‘Valencia’ orange were shown in Fig 5 and there were no any abnormalities except for the presence of one quadrivalent at diakinesis. The chromosome conjugation at diakinesis of ‘Valencia’ had been statistical analysis and the results showed that the number of bivalents ranged from 7 to 9, with 19.8% of the PMCs showing 9 bivalents (Fig 5B), and 79.2% of the PMCs had 7 bivalents (Fig 5C). Presence of 9 bivalents indicated homology between the parental genomes. Presence of 7 bivalents and one quadrivalent showed that except for four chromosomes, the other 14 chromosomes were homologous to each other. The quadrivalent showed by the arrow in Fig 5C duly indicates the occurrence of a reciprocal translocation.

## Discussion

### Cytological heterozygosity of in ‘Valencia’ sweet orange (*C*. *sinensis*)

**Fig 6:**
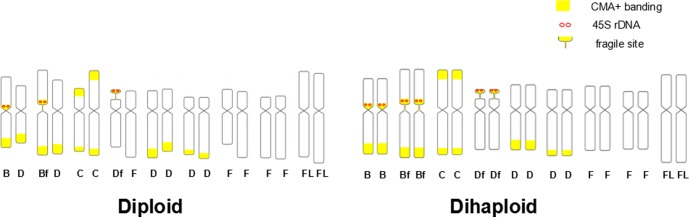
Idiogram of *C*. *sinensis* chromosomes with CMA+ bands, 45S rDNA sites and fragile sites. Letters Bf, B, C, Df, D and F indicate chromosome types. Large yellow blocks represent CMA+ bands. Yellow short vertical lines suggest fragile sites and red small circles indicate 45S r DNAs co-localized with CMA+ bands.

The combination of chromosome morphology, CMA+ banding and FISH with tandem repeat DNA probes enables the distinguishing of many chromosome types in *C*. *sinensis* and its dihaploid line (Fig 6). The CMA/DAPI banding patterns together with the FISH results observed in *C*. *sinensis* in the present study are basically consistent with those previously reported [6, 33, 38, 39]. Here, it was further suggested that there were two fragile sites respectively observed on B-type and D-type chromosomes (namely Bf and Df in this study). Taking the fragile sites into consideration, the karyotype formula of *C*.*sinensis* was modified as B + Bf + 2C + Df + 6D + 7F.

Pedrosa et al [10] and Barros e Silva et al [31] performed CMA/DAPI banding and FISH with rDNA and the results showed the two B-type chromosomes are homologous chromosomes and the D-type chromosome with 45S rRNA was homologous with one F-type chromosome. No difference in CMA bands and 45S rRNA was observed in original parent diploid *C*.*sinensis*. According to the karyotypeformula of the original parent diploid plants, the karyotype formula of doubled haploid line obtained by anther culture should have been 2B + 2C + 6D + 8F or 2B + 2C + 8D + 6F. However, the results of chromosomal characterization in doubled haploid were out of our expectation. In the present work, the callus of doubled haploid showed a karyotype formula of 2B + 2Bf + 2C + 2Df + 4D + 6F and presented the same CMA-positive bands and signals of all DNA sequences, which is in contrast with the chromosomal characteristics of the original parent diploid plants. Furthermore, there were six sites of 45S rDNA in doubled haploid, with two pairs proximally located on B-type and Bf-type chromosomes and one pair subterminally located on Df chromosomes, which had been further supported by the genome sequencing of *C*. *sinensis* [2]. ‘Valencia’ was reported to be a heterozygous reciprocal translation which resulted in meiotic irregularities [40]. It is possible that B-type and Bf-type were homeologous chromosomes, which were always considered to be homologous as previously reported [8, 31, 41]. We speculated reciprocal translation between the Bf-type chromosome and one D chromosome occurred and then formed a new D-type and Bf-type chromosomes, which together with other B-type and D-type chromosomes form quadrivalent (Fig 5C). Chromosome rearrangements are probably restricted to fragile site of Bf-type chromosome, which resulted in no difference of karyogram in different cultivars of *C*.*sinensis* and reasonably explained the change in doubled haploid (Fig 6, dihaploid). There may be another possibility that B and Bf chromosomes are not homologous showed by the idiogram of *C*. *sinensis* (Fig 6, diploid). The haploid inherited the chromosomes harboring 45s rDNA from the parental diploid, which were then doubled in the doubled haploid further supported by 9 bivalents chromosome conjugation at diakinesis of ‘Valencia’ (Fig 5B).

To our knowledge, this is the first report that doubled haploid callus line was used for cytological research in *Citrus*. Chromosomal characteristics of *C*. *sinensis* and its doubled haploid revealed by CMA/DAPI banding and repetitive DNAs FISH clearly illuminate that *C*. *sinensis* is a high heterozygosis as reported in previous studies, and doubled haploid appears to be very important for the identification of heterozygosity in *Citrus*. *C*. *sinensis* is characterized by chromosomal heterozygosity, as evidenced by the fact that it is comparatively difficult to recognize and pair homologous chromosomes through DAPI-CMA banding and 45S rDNA localization analysis [8]. For example, the B-type and the Bf-type chromosomes were considered to be a pair of homologous chromosomes absolutely; the two C-type chromosomes differed from each other in size of chromosomes and CMA+ bands; and the homologous chromosomes of Df chromosome could not be found. The factors contributing to chromosomal heterozygosity possibly include the origin of interspecific hybridization and clonal propagation which allows accumulation of karyotypic rearrangements [8]. The difference of homologous chromosomes could be mainly explained by the amplification and reduction of the satellite DNA sequences or introgression to different extents [12–14].

### Characterization of fragile sites in *C*. *sinensis*

During the course of mapping the rDNA in *C*. *sinensis*, we discovered some chromosome breaks and gaps, which were all observed exclusively in the 45S rDNA sites in meristematic cells. The characteristics of chromosome breakages or gaps observed in this study are cytologically very consistent with the features of fragile sites observed in the chromosomes of human and *Lolium* [19, 27, 29, 30, 42]. In human chromosomes, fragile sites could not be seen through routine cytological observations and appeared as complete breaks, decondensed chromatins or fibers [43, 44]. In the chromosomes of *Lolium*, 45S rDNA regions as the chromosome fragile sites were frequently reported [27–30]. In our study, chromosomes fragile sites of sweet orange were confirmed by fluorescence staining, telomeric DNA and 45S rDNA FISH, which also could not be recognized through single routine cytological method. This unusual and interesting phenomenon in *Citrus* led us to the effort of characterizing these chromosome breakages or gaps associated with rDNAs and exploring the possible functions of fragile sites.

In this study, CMA banding and FISH using 45S rDNA as a probe revealed that there were two forms of 45s rDNA signals. One form of 45s rDNA loci was extended segments, while the other form appeared to be in a condensed state (Fig 3). Extended 45S rDNA segments showed a depleted or decondensed chromatin structure during metaphase, which looked like a few thin threads joining together. Ag-NOR staining revealed that every cell had two NORs, and the two decondensed 45S rDNA sites had transcriptional activity. High concentrations of heavily transcribed rDNA genes are toxic to the cells of yeast, which interferes with cohesion between rDNA loci of sister chromatids leading to genome instability [16]. FISH and Ag-NOR revealed that gaps occurred exclusively at the 45S rDNA sites, and the subterminal one was on D-type chromosome and the proximal one was on B-type chromosome. From these results, we conclude, for the first time in *C*. *sinensis* that there are only two chromosome fragile sites present either as gaps or breaks on metaphase chromosomes and it always located on proximal B-type chromosome and on subteminal D-type chromosome. The fragile sites were inherited steadily in *C*. *sinensis* and doubled in its double haploid, which indicated the fragile sites were non-random and heritable reported in genetically modified maize [45].

In many previous studies, it has been suggested that fragile sites are non-randomly localized in 45s rDNA regions and could occur randomly between multiple 45S rDNA regions, and distended 45S rDNAs are observed to be transcriptionally active in *Phleum* and *Lolium* [29, 30, 46]. Distended loci were found to be hypo-methylated, while the silenced condensed site was strongly 5-mC methylated in *C*.*sinensis*; and there was a positive correlation between the condensation state of 45S rDNA sites and the level of DNA methylation [18]. Different condensed types of 45S rDNA sites in *Jatropha curcas* L. appeared different levels of DNA methylation which was considered to be an epigenetic marker for rDNA activation [47]. In conclusion, the distended loci in *C*.*sinensis* observed by Marques et al [18] were fragile sites, which were active and hypo-methylated, whereas methylation of CpG dinucleotides at or near these fragile sites enhances the fragile phenotype [25].

### Characterization of fragile sites in *Citrus* and related genera

Fragile sites are manifested as non-random incomplete breaks or gaps with chromatin fibers on prometaphase and metaphase chromosomes [48, 49]. So far, chromosome lesions caused by the occurrence of fragile sites have been most frequently observed in human [43]. In plants, chromosome breaks are definitely less frequent and almost exclusively restricted 45S rDNA sites, which have been analyzed in more detail in *Lolium*. Cytologically, the characteristics of the gaps co-localized with 45s rDNAs observed in this study are in agreement with those of the fragile sites observed in *Lolium* chromosomes [27, 29, 30]. This is the first report dealing with the characterization of chromosome breakages in *C*. *sinensis* on metaphase chromosomes. In this investigation, we reported that the distended 45S rDNA loci as the chromosome fragile sites were spontaneously expressed *in vitro* on metaphase in *Citrus* and related genera. The D-type chromosome with highly repeated satellite DNA (stDNA) was often named as Dst in karyotype analysis [50], which cytologically resembles the chromosome Df.

Fragile sites were found to be located at very end of D-type chromosome and proximal to B-type chromosome in *C*. *sinensis*, and were doubled in its doubled haploid line. Based on the previously reported karyotype formulas of *Citrus* and related genera [8, 31–35], the regions present as gaps, constrictions and breaks lightly–stained by DAPI/CMA, and were considered to be fragile sites. We speculate that there are four different types of fragile sites in *Citrus* and related genera species: (1) Af (fragile site at the proximal CMA^+^ band of A-type chromosome, *C*.*maxima*); (2) Bf (fragile site at the proximal CMA^+^ band of B- type chromosome, *C*. *medica*); (3) Cf (fragile site at one of subterminal CMA^+^ bands of C- type chromosome, *S*. *buxifolia*); (4) Df (fragile site at the subterminal CMA^+^ band of D-type chromosome, *C*.*micrantha*), which is suggested to be the most common fragile site. Although the types of fragile sites in *Citrus* and its genera vary, the number of fragile sites keeps relatively constant. There are two fragile sites in majority of species but three in pomelo (*C*. *maxima*). Hexaploid *G*. *pentaphylla* has six fragile sites, which may be within the normal range. The exception here is the doubled haploid of *C*. *sinensis*, which has four fragile sites. The callus line of ‘Valencia’ derived from anther culture could not generate plantlet [4], while the plants regenerated from two doubled haploid lines of 'Early Gold' sweet orange grew vigorously in the greenhouse[51], suggesting that some recessively harmful genes are expressed after homozygosity [52], or redundant fragile sites lead to genomic instability. Common fragile sites in human genome are particularly prone to instability under conditions of replicative stress [44]. Two more fragile sites in the dihaploid line of ‘Valencia’ orange we discovered would cause the instability of the genome. However, how the increase of fragile sites influences regeneration should be further investigated. The roles of fragile sites have been widely discussed, resulting in conflicting findings in different organisms. Human fragile sites are hotspots for chromosome rearrangements [24, 53–55], and are often associated with sequence/structural motifs that hinder the DNA replication fork [56]. Incidence of fragile sites may be involved in the process of chromosomal instability (such as rearrangements and amplifications), which constitutes a potential mechanism for speciation [57, 58]. Various types and constant number of fragile sites in *Citrus* and related genera may be associated with karyotype evolution and the formation of new species. Yet, the roles of fragile sites in evolution still remain elusive. Thus, further researches are required to know the more detailed functions of fragile sites.

## References

[1] Talon M, Gmitter FG Jr (2008) *Citrus* genomics. Int J Plant Genomics 2008:1–17. 10.1155/2008/528361 PMC2396216 18509486

[2] Xu Q, Chen LL, Ruan X, Chen D, Zhu A, Chen CL, et al (2013) The draft genome of sweet orange (*Citrus sinensis*). Nat Genet 45(1):59–66. 10.1038/ng.2472 23179022

[3] Scora RW (1975) On the history and origin of *Citrus*. Bull. Torrey Bot. Club 102(12):369–375.

[4] Cao H, Biswas MK, Lü Y, Amar MH, Tong Z, Xu Q, et al (2011) Doubled haploid callus lines of Valencia sweet orange recovered from anther culture. Plant Cell Tiss Organ Cult 104(3):415–423.

[5] Carvalho R, Soares Filho WS, Brasileiro-Vidal AC, Guerra M (2005) The relationships among lemons, limes and citron a chromosomal comparison. Cytogenet Genome Res 109(1–3):276–282. 15753587 10.1159/000082410

[6] Guerra M (1993) Cytogenetics of Rutaceae. V. High chromosomal variability in *Citrus* species revealed by CMA/DAPI staining. Heredity 71(3):234–241.

[7] Cornélio MTMN, Figueirôa ARS, Santos KGB, Carvalho R, Soares Filho WS, Guerra M (2003) Chromosomal relationships among cultivars of *Citrus reticulata* Blanco, its hybrids and related species. Plant Syst Evol 240(1):149–161.

[8] Moraes AP, Soares Filho WS, Guerra M (2007) Karyotype diversity and the origin of grapefruit. Chromosome Res 15(1):115–121. 17295131 10.1007/s10577-006-1101-2

[9] Younis A, Ramzan F, Hwang YJ, Lim KB (2015) FISH and GISH: molecular cytogenetic tools and their applications in ornamental plants. Plant Cell Rep 34(9):1477–1488. 10.1007/s00299-015-1828-3 26123291

[10] Pedrosa A, Schweizer D, Guerra M (2000) Cytological heterozygosity and the hybrid origin of sweet orange [*Citrus sinensis* (L.) Osbeck]. Theor Appl Genet 100(3–4):361–367.

[11] Costa Silva S, Marques A, Soares Filho WS, Mirkov TE, Pedrosa-Harand A, Guerra M (2011) The cytogenetic map of the *Poncirus trifoliata* (L.) Raf.—a nomenclature system for chromosomes of all citric species. Trop Plant Biol 4(2):99–105.

[12] Costa Silva S, Mendes S, Soares Filho WS, Pedrosa-Harand A (2015) Chromosome homologies between *Citrus* and *Poncirus*—the comparative cytogenetic map of mandarin (*Citrus reticulata*). Tree Genet Genomes 11:811

[13] Mendes S, Moraes AP, Mirkov TE, Pedrosa-Harand A (2011) Chromosome homeologies and high variation in heterochromatin distribution between *Citrus* L. and *Poncirus* Raf. as evidenced by comparative cytogenetic mapping. Chromosome Res 19(4):521–530. 10.1007/s10577-011-9203-x 21468689

[14] Moraes AP, Mirkov TE, Guerra M (2008) Mapping the chromosomes of *Poncirus trifoliata* Raf. by BAC-FISH. Cytogenet Genome Res 121(3–4):277–281. 10.1159/000138897 18758171

[15] Dvorackova M, Fojtova M, Fajkus J (2015) Chromatin dynamics of plant telomeres and ribosomal genes. Plant J 83(1):18–37. 10.1111/tpj.12822 25752316

[16] Ide S, Miyazaki T, Maki H, Kobayashi T (2010) Abundance of ribosomal RNA gene copies maintains genome integrity. Science 327(5966):693–696. 10.1126/science.1179044 20133573

[17] Chung MC, Lee YI, Cheng YY, Chou YJ, Lu CF (2008) Chromosomal polymorphism of ribosomal genes in the genus *Oryza*. Theor Appl Genet 116(6):745–753. 10.1007/s00122-007-0705-z 18214422 PMC2271086

[18] Marques A, Fuchs J, Ma L, Heckmann S, Guerra M, Houben A (2011) Characterization of eu- and heterochromatin of C*itrus* with a focus on the condensation behavior of 45S rDNA chromatin. Cytogenet Genome Res 134(1):72–82. 10.1159/000323971 21304248

[19] Richards RI (2001) Fragile and unstable chromosomes in cancer causes and consequences. Trends Genet 17(6):339–345. 11377796 10.1016/s0168-9525(01)02303-4

[20] Robert-Fortel I, Junéra HR, Géraud G, Hernandez-Vcrdun D (1993) Three-dimensional organization of the ribosomal genes and Ag-NOR proteins during interphase and mitosis in PtK1 cells studied by confocal microscopy. Chromosoma 102(3):146–157. 7681367 10.1007/BF00387729

[21] Tchurikov NA, Fedoseeva DM, Sosin DV, Snezhkina AV, Melnikova NV, Kudryavtseva AV, et al (2015) Hot spots of DNA double-strand breaks and genomic contacts of human rDNA units are involved in epigenetic regulation. J Mol Cell Biol 7 (4):366–382. 10.1093/jmcb/mju038 25280477 PMC4524424

[22] Rosen DM, Younkin EM, Miller SD, Casper AM (2013) Fragile site instability in *Saccharomyces cerevisiae* causes loss of heterozygosity by mitotic crossovers and break-induced replication. PLoS Genet 9(9): 119–129. 10.1371/journal.pgen.1003817 PMC3778018 24068975

[23] Debacker K, Kooy RF (2007) Fragile sites and human disease. Hum Mol Genet 16 Review Issue 2:R150- R 158. 10.1093/hmg/ddm136 17567780

[24] Hellman A, Zlotorynski E, Scherer SW, Cheung J, Vincent JB, Smith DI, et al (2002) A role for common fragile site induction in amplification of human oncogenes. Cancer Cell 1(1):89–97. 12086891 10.1016/s1535-6108(02)00017-x

[25] Wang Y-H, Griffith J (1996) Methylation of expanded CCG triplet repeat DNA from fragile X syndrome patients enhances nucleosome exclusion. J Biol Chem 271(38):22937–22940. 8798475

[26] Wang Y-H (2006) Chromatin structure of human chromosomal fragile sites. Cancer Lett 232(232):70–78. 16229940 10.1016/j.canlet.2005.07.040

[27] Huang J, Ma L, Yang F, Fei S-z, Li L (2008) 45S rDNA regions are chromosome fragile sites expressed as gaps *in vitro* on metaphase chromosomes of toot-tip meristematic cells in *Lolium* spp. PLoS One 3(5):e2167 10.1371/journal.pone.0002167 18478113 PMC2366065

[28] Huang J, Ma L, Sundararajan S, Fei S-z, Li L (2009) Visualization by atomic force microscopy and FISH of the 45S rDNA gaps in mitotic chromosomes of *Lolium perenne*. Protoplasma 236(1–4):59–65. 10.1007/s00709-009-0051-x 19468820

[29] Bustamante FO, Rocha LC, Torres GA, Davide LC, Mittelmann A, Techio VH (2014) Distribution of rDNA in diploid and polyploid *Lolium multiflorum* Lam.and fragile sites in 45S rDNA regions. Crop Sci 54:617–625. Rocha LC, Bustamante FO, Silveira RAD, Torres GA, MittelmannA, Techio VH (2015) Functional repetitive sequences and fragile sites in chromosomes of *Lolium perenne* L. Protoplasma 252:451–460. 10.1007/s00709-014-0690-4 25141824

[30] Huang M, Li H, Zhang L, Gao F, Wang P, Hu Y, et al (2012) Plant 45S rDNA clusters are fragile sites and their instability is associated with epigenetic alterations. PLoS One 7(4):e35139 10.1371/journal.pone.0035139 22509394 PMC3324429

[31] Barros e Silva AE, Marques A, dos Santos KGB, Guerra M (2010) The evolution of CMA bands in *Citrus* and related genera. Chromosome Res 18(4):503–514. 10.1007/s10577-010-9130-2 20490650

[32] Barros e Silva AE, Soares Filho WS, Guerra M (2013) Linked 5S and 45S rDNA sites are highly conserved through the subfamily Aurantioideae (Rutaceae). Cytogenet Genome Res 140(1):62–69. 10.1159/000350695 23635472

[33] Guerra M, Santos KGB, Barros e Silva AE, Ehrendorfer F (2000) Heterochromatin banding patterns in Rutaceae-Aurantioi-deae–a case of parallel chromosomal evolution. Am J Bot 87(5):735–747. 10811798

[34] Yamamoto M, Asadi Abkenar A, Matsumoto R, Nesumi H, Yoshida T, Kuniga T, et al (2007) CMA banding patterns of chromosomes in major *Citrus* species. J. Japan. Soc. Hort. Sci 76(1):36–40.

[35] Yamamoto M, Asadi Abkenar A, Matsumoto R, Kubo T, Tominaga S (2008) CMA staining analysis of chromosomes in several species of Aurantioideae. Genet Resour Crop Ev 55(55):1167–1173.

[36] Kang L, Du X, Zhou Y, Zhu B, Ge X, Li Z (2014) Development of a complete set of monosomic alien addition lines between *Brassica napus* and *Isatis indigotica* (Chinese woad). Plant Cell Rep 33(8):1355–1364. 10.1007/s00299-014-1621-8 24781060

[37] Cui C, Ge X, Gautam M, Kang L, Li Z (2012) Cytoplasmic and genomic effects on meiotic pairing in *Brassica* hybrids and allotetraploids from pair crosses of three cultivated diploids. Genetics 191(3):725–738. 10.1534/genetics.112.140780 22505621 PMC3389969

[38] Miranda M, Ikeda F, Endo T, Moriguchi T, Omura M (1997) Comparative analysis on the distribution of heterochromatin in *Citrus*, *Poncirus* and *Fortunella* chromosomes. Chromosome Res 5(2):86–92. 9146911 10.1023/a:1018409922843

[39] Matsuyama T, Akihama T, Ito Y, Omura M, Fukui K (1996) Characterization of heterochromatic regions in 'Trovita’ orange (*Citrus sinensis* Osbeck) chromosomes by the fluorescent staining and FISH methods. Genome 39(5):941–945. 8890521 10.1139/g96-118

[40] Iwamasa M (1966) Studies on the sterility in genus *Citrus* with special reference to the seedlessness. Bul Hort Res Sta B6:1–81.

[41] Lu X, Zhou W, Gao F (2010) Chromosomal location of 45S rDNA and *dfr* gene in *Citrus sinensis*. Biol Plant 54(4):798–800.

[42] Glover TW (2006) Common fragile sites. Cancer Lett 232(1):4–12. 16229941 10.1016/j.canlet.2005.08.032

[43] Sbrana I, Zavattari P, Barale R, Musio A (1998) Common fragile sites on human chromosomes represent transcriptionally active regions: evidence from camptothecin. Hum Genet 102(4):409–414. 9600236 10.1007/s004390050713

[44] Cimprich KA (2003) Fragile sites: breaking up over a slowdown. Curr Biol 13(6):R231–R233. 12646149 10.1016/s0960-9822(03)00158-1

[45] Waminal NE, Ryu KH, Choi S-H, Kim HH (2013) Randomly detected genetically modified (GM) maize (*Zea mays* L.) near a transport route revealed a fragile 45S rDNA phenotype. PLoS One 8(9): e74060 10.1371/journal.pone.0074060 24040165 PMC3767626

[46] Grabowska-Joachimiak A, Kula A, Gernand-Kliefoth D, Joachimiak AJ (2015) Karyotype structure and chromosome fragility in the grass *Phleum echinatum* Host. Protoplasma 252(1):301–306. 10.1007/s00709-014-0681-5 25056831 PMC4287660

[47] Gong Z, Xue C, Zhang M, Guo R, Zhou Y, Shi G (2013) Physical localization and DNA methylation of 45S rRNA gene loci in *Jatropha curcas* L. PLoS One 8(12):e84284 10.1371/journal.pone.0084284 24386362 PMC3875529

[48] Casper AM, Nghiem P, Arlt MF, Glover TW (2002) ATR regulates fragile site stability. Cell 111(6):779–789. 12526805 10.1016/s0092-8674(02)01113-3

[49] Harrison CJ, Jack EM, Allen TD, Harris R (1983) The fragile X: a scanning electron microscope study. J Med Genet 20(4):280–285. 6684694 10.1136/jmg.20.4.280 PMC1049120

[50] Fann JY, Kovarik A,·Hemleben V, Tsirekidze NI, Beridze TG (2001) Molecular and structural evolution of *Citrus* satellite DNA. Theor Appl Genet 103(6–7):1068–1073.

[51] Wang SM, Lan H, Cao HB, Xu Q, Chen CL, Deng XX, et al (2015) Recovery and characterization of homozygous lines from two sweet orange cultivars *via* anther culture. Plant Cell Tiss Organ Cult 123(3):1–12.

[52] Deng XX, Deng ZA, Xiao SY, Zhang WC (1992) Pollen derived plantlets from anther culture of Ichang papeda hybrids No.14 and Trifoliate orange. In: Proceedings of the 7th International Citrus Congress, Acireale, Italy, March 1992. International Society of Citriculture, pp 190–192.

[53] Gao G, Smith DI (2014) Very large common fragile site genes and their potential role in cancer development. Cell Mol Life Sci 71(23):4601–4615. 10.1007/s00018-014-1753-6 25300511 PMC11113612

[54] Ozeri-Galai E, Tur-Sinai M, Bester AC, Kerem B (2014) Interplay between genetic and epigenetic factors governs common fragile site instability in cancer. Cell Mol Life Sci 71(23):4495–4506. 10.1007/s00018-014-1719-8 25297918 PMC11113459

[55] Schwartz M, Zlotorynski E, Goldberg M, Ozeri E, Rahat A, Sage C, et al (2005) Homologous recombination and nonhomologous end-joining repair pathways regulate fragile site stability. Genes Dev 19(22):2715–2726. 16291645 10.1101/gad.340905 PMC1283964

[56] Song W, Dominska M, Greenwell PW, Petes TD (2014) Genome-wide high-resolution mapping of chromosome fragile sites in *Saccharomyces cerevisiae*. P Natl Acad Sci USA 111(21):E2210–E2218. 10.1073/pnas.1406847111 PMC4040566 24799712

[57] Brown JD, O'Neill RJ (2010) Chromosomes, conflict, and epigenetics: chromosomal speciation revisited. Annu Rev Genomics Hum Genet 11:291–316. 10.1146/annurev-genom-082509-141554 20438362

[58] Ruiz-Herrera A, Robinson TJ (2007) Chromosomal instability in Afrotheria: fragile sites, evolutionary breakpoints and phylogenetic inference from genome sequence assemblies. BMC Evol Biol 7(1):1–15. 17958882 10.1186/1471-2148-7-199 PMC2211313

